# 7-Bromo-4b-methyl-7,8-dihydro-4b*H*-9-thia-8a-aza­fluorene 9,9-dioxide

**DOI:** 10.1107/S1600536808017972

**Published:** 2008-06-19

**Authors:** Judith C. Gallucci, Robert D. Dura, Leo A. Paquette

**Affiliations:** a Evans Chemical Laboratories, Ohio State University, 100 West 18th Avenue, Columbus, OH 43210, USA

## Abstract

The title compound, C_12_H_12_BrNO_2_S, was isolated after direct irradiation (hν 350 nm, hexa­ne) of a mixture of stereoisomeric sulfonamides containing a vicinal dibromide and a conjugated diene. This product is one of a group of substrates that has contributed to our understanding of the photoreactivity patterns of non-bridged sulfonamides. The crystal structure was determined from a non-merohedrally twinned data set, where the twin law corresponded to a 180° rotation about the a* axis. The minor twin component refined to a value of 0.176 (3). The conformation of the mol­ecule is planar at one end, as the benzene ring and the adjacent fused five-membered ring are coplanar, and U-shaped at the other end, where the five-membered ring is fused to the heterocyclic six-membered ring containing an allyl bromide group.

## Related literature

For related chemistry, see: Dura & Paquette (2006 ▶); Paquette *et al.* (2004 ▶, 2006 ▶). For related literature, see: Cooper *et al.* (2002 ▶).
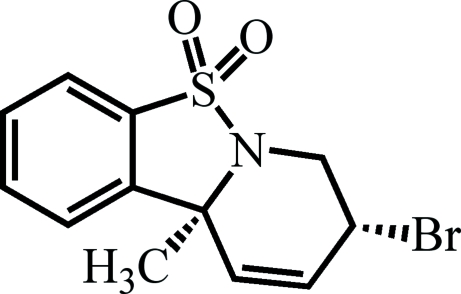


## Experimental

### 

#### Crystal data

C_12_H_12_BrNO_2_S
*M* *_r_* = 314.2
Monoclinic, 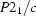

*a* = 14.3970 (3) Å
*b* = 7.8912 (1) Å
*c* = 11.4652 (2) Å
β = 103.009 (1)°
*V* = 1269.13 (4) Å^3^
*Z* = 4
Mo *K*α radiation
μ = 3.39 mm^−1^
*T* = 293 (2) K
0.38 × 0.27 × 0.04 mm

#### Data collection

Nonius KappaCCD diffractometer
Absorption correction: multi-scan (*SCALEPACK*; Otwinowski & Minor, 1997 ▶) *T*_min_ = 0.650, *T*_max_ = 0.873
18122 measured reflections
2835 independent reflections
2401 reflections with *I* > 2σ(*I*)
*R*_int_ = 0.055

#### Refinement

*R*[*F* ^2^ > 2σ(*F* ^2^)] = 0.044
*wR*(*F* ^2^) = 0.115
*S* = 1.49
1509 reflections
156 parameters
H-atom parameters constrained
Δρ_max_ = 0.62 e Å^−3^
Δρ_min_ = −0.29 e Å^−3^

### 

Data collection: *COLLECT* (Nonius, 2000 ▶); cell refinement: *SCALEPACK* (Otwinowski & Minor, 1997 ▶); data reduction: *DENZO* (Otwinowski & Minor, 1997 ▶) and *SCALEPACK*; program(s) used to solve structure: *SHELXS97* (Sheldrick, 2008 ▶); program(s) used to refine structure: *SHELXL97* (Sheldrick, 2008 ▶); molecular graphics: *ORTEP-3 for Windows* (Farrugia, 1997 ▶); software used to prepare material for publication: *WinGX* (Farrugia, 1999 ▶).

## Supplementary Material

Crystal structure: contains datablocks global, I. DOI: 10.1107/S1600536808017972/rt2018sup1.cif

Structure factors: contains datablocks I. DOI: 10.1107/S1600536808017972/rt2018Isup2.hkl

Additional supplementary materials:  crystallographic information; 3D view; checkCIF report

## Figures and Tables

**Fig. 1.**
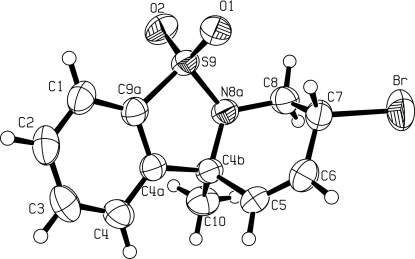
The molecular structure of (I) is drawn with 50% probability displacement ellipsoids for non-hydrogen atoms. The hydrogen atoms are drawn with an artificial radius.

**Fig. 2.**
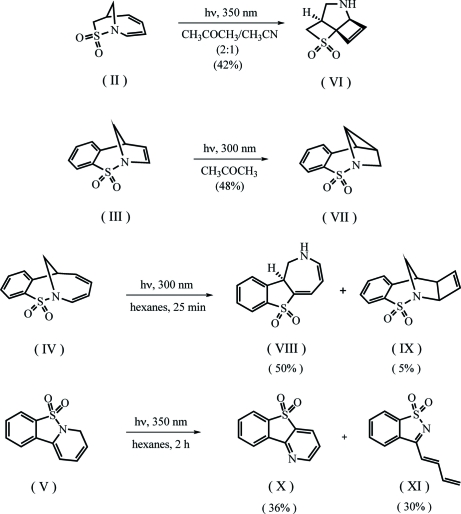
Photochemical reactions for sulfonamides (II—V).

**Fig. 3.**
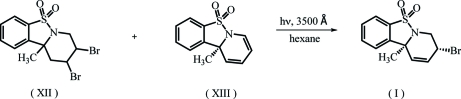
Photosynthesis of (I) from (XII) and (XIII).

## supplementary crystallographic information

### Comment

The crystal structure of compound (I), C_12_H_12_BrNO_2_S, was determined as part of a study of the photoreactivity of non-bridged sulfonamides and is shown in Fig. 1.

The photo-induced direct or triplet-sensitized irradiation of sulfonamides (II), (III), (IV) and (V) have established that the proximity of a sulfonamide functionality to a conjugated diene unit can have one of several chemical consequences (Paquette *et al.*, 2004, 2006; Dura & Paquette, 2006) as shown in Fig. 2. Whereas previous studies have focused on bicyclic frameworks with the sulfonamide nitrogen occupying a bridgehead position, *e.g.* (II), (III) and (IV), the fourth compound in this series, (V), was designed to be considerably more planar in nature for the purpose of probing the relationship between photoreactivity and nitrogen geometry. The title compound is the product of a study currently underway to assess the consequences of the more planar nature of these heterocyclic sulfonamides containing a diene unit.

Compound (I) was produced photochemically from a mixture of compounds (XII) and (XIII) as shown in Fig. 3. Unlike the previous sulfonamides depicted in Fig. 2, (XIII) has proven to be resistant per se to inter- or intramolecular photochemical transformations. However, an equimolar mixture of the vicinal dibromide (XII) and the diene (XIII) has been observed to yield the title compound (I). The mechanism of this transformation is currently under investigation.

One end of molecule (I) is planar, as the benzene ring and adjacent five-membered ring, consisting of atoms S9, N8a, C4b, C4a and C9a, are essentially coplanar. The other end of the molecule can be described as U-shaped when considering the same five-membered ring combined with the heterocyclic six-membered ring containing the allyl bromide. The conformation of this heterocyclic ring is such that atoms N8a, C4b, C5, C6 and C7 are approximately coplanar, with atom C8 lying 0.620 (5) Å out of this plane. The nitrogen atom is pyramidal and its lone pair of electrons is on the same side of the molecule as the methyl group. The methyl and Br groups are oriented *cis* with respect to each other.

This structure was determined from a non-merohedrally twinned data set. The twin law corresponded to a 180° rotation about the a* axis, and was obtained with the aid of the Rotax program (Cooper *et al.*, 2002). Application of this twin law to the data to create an HKLF 5 format data set (Sheldrick, 2008) was done with the *WinGX* software (Farrugia, 1999). The minor twin component refined to a value of 0.176 (3).

### Experimental

A mixture of the vicinal dibromide (XII) (8.4 mg, 0.021 mmol) and diene (XIII) (5.0 mg, 0.021 mmol) was dissolved in hexane/dichloromethane (3:2), deoxygenated with Ar for 1 h, and placed in a quartz reaction vessel. That mixture was then irradiated with a bank of 3500 Å lamps for 1.5 h, concentrated, and purified by means of preparative basic alumina plate chromatography (elution with dichloromethane) to provide 1.1 mg of the title compound (I) (17%). Colorless crystals of (I) were grown from chloroform and have a melting point of 148–150°C.

### Refinement

During the final stages of the refinement before application of the twin law, the *R* factors were slightly large, with R1 = 0.075 for I>2σ(I), the largest peak in the difference electron density map was 0.92 e/Å^3^, and the errors in the metrical parameters were large, with a C—C bond length error typically around 0.01 Å. Since the precession images assembled from the data frames indicated that twinning was a possibility, Rotax (Cooper *et al.*, 2002) was used to generate various twin laws. The twin law corresponding to a 180° rotation about the a* axis was applied to the data set, since it agreed with the appearance of the precession images. This twin law in matrix form is as follows: [1 0 0.565 / 0 -1 0 / 0 0 -1]. Application of this twin law to the data to create an HKLF 5 format data set (Sheldrick, 2008) was done with the *WinGX* software (Farrugia, 1999). Since this is a non-merohedral twin, it was necessary to generate and test several data sets based on different overlap criteria for the reflections. The set of overlap criteria which minimized the *R* factors was chosen as the best set.

The final results are based on this modified data set, which contains composite reflections (reflections exactly overlapped due to twinning) and single reflections. Still other reflections are omitted from the data set because they are partially overlapped with their twin component reflections and could not be integrated with the data integration software. As a result, the completeness of the data set is low at 67.7%. The minor twin component refined to a value of 0.176 (3). Application of this twin law to the data set has resulted in an overall improvement in the model with a decrease in the *R* values, a decrease in the largest peak in the final difference map and a decrease in the errors in the metrical parameters.

### Crystal data

**Table:**

C_12_H_12_Br_1_N_1_O_2_S_1_	*F*_000_ = 632
*M**_r_* = 314.2	*D*_x_ = 1.644 Mg m^−^^3^
Monoclinic, *P*2_1_/*c*	Mo *K*α radiation λ = 0.71073 Å
Hall symbol: -P 2ybc	Cell parameters from 2393 reflections
*a* = 14.3970 (3) Å	θ = 2.0–25.0º
*b* = 7.8912 (1) Å	µ = 3.39 mm^−^^1^
*c* = 11.4652 (2) Å	*T* = 293 (2) K
β = 103.009 (1)º	Plate, colorless
*V* = 1269.13 (4) Å^3^	0.38 × 0.27 × 0.04 mm
*Z* = 4	

### Data collection

**Table:**

Nonius KappaCCD diffractometer	2835 independent reflections
Monochromator: graphite	2401 reflections with *I* > 2σ(*I*)
Detector resolution: 9 pixels mm^-1^	*R*_int_ = 0.055
*T* = 293(2) K	θ_max_ = 25.0º
φ and ω scans	θ_min_ = 2.9º
Absorption correction: multi-scan(SCALEPACK; Otwinowski & Minor, 1997)	*h* = −17→16
*T*_min_ = 0.650, *T*_max_ = 0.873	*k* = −9→9
18122 measured reflections	*l* = −13→13

### Refinement

**Table:**

Refinement on *F*^2^	Secondary atom site location: difference Fourier map
Least-squares matrix: full	Hydrogen site location: inferred from neighbouring sites
*R*[*F*^2^ > 2σ(*F*^2^)] = 0.044	H-atom parameters constrained
*wR*(*F*^2^) = 0.115	*w* = 1/[σ^2^(*F*_o_^2^) + (0.0519*P*)^2^ + 1.4699*P*] where *P* = (*F*_o_^2^ + 2*F*_c_^2^)/3
*S* = 1.49	(Δ/σ)_max_ = 0.001
1509 reflections	Δρ_max_ = 0.62 e Å^−^^3^
156 parameters	Δρ_min_ = −0.29 e Å^−^^3^
Primary atom site location: structure-invariant direct methods	Extinction correction: none

### Special details

**Table:**

Experimental. Examination of the diffraction pattern on a Nonius Kappa CCD diffractometer indicated a monoclinic crystal system. All work was done at room temperature. The data collection strategy was set up to measure a quadrant of reciprocal space with a redundancy factor of 3.7, which means that 90% of the reflections were measured at least 3.7 times. Phi and omega scans with a frame width of 1.0 degree were used for data collection. Data integration was done with *DENZO* (Otwinowski & Minor, 1997) and scaling and merging of the data was done with *SCALEPACK* (Otwinowski & Minor, 1997).Structure solution was done by a combination of the Patterson method and the direct methods procedure in *SHELXS97* (Sheldrick, 2008). Full-matrix least-squares refinements based on F^2^ were performed in *SHELXL97* (Sheldrick, 2008), as incorporated in the *WinGX* package (Farrugia, 1999).
Geometry. All e.s.d.'s (except the e.s.d. in the dihedral angle between two l.s. planes) are estimated using the full covariance matrix. The cell e.s.d.'s are taken into account individually in the estimation of e.s.d.'s in distances, angles and torsion angles; correlations between e.s.d.'s in cell parameters are only used when they are defined by crystal symmetry. An approximate (isotropic) treatment of cell e.s.d.'s is used for estimating e.s.d.'s involving l.s. planes.
Refinement. For the methyl group, the hydrogen atoms were added at calculated positions using a riding model with C—H=0.96 Å and U(H)=1.5**U*_eq_(bonded carbon atom). The torsion angle, which defines the orientation of the methyl group about the C—C bond, was refined. The remaining hydrogen atoms were included in the model at calculated positions using a riding model with a range of C—H distances from 0.93 to 0.98 Å and U(H)=1.2**U*_eq_(bonded carbon atom).Refinement of *F*^2^ against ALL reflections. The weighted *R*-factor *wR* and goodness of fit *S* are based on *F*^2^, conventional *R*-factors *R* are based on *F*, with *F* set to zero for negative *F*^2^. The threshold expression of *F*^2^ > σ(*F*^2^) is used only for calculating *R*-factors(gt) *etc*. and is not relevant to the choice of reflections for refinement. *R*-factors based on *F*^2^ are statistically about twice as large as those based on *F*, and *R*- factors based on ALL data will be even larger.

### Fractional atomic coordinates and isotropic or equivalent isotropic displacement parameters (Å^2^)

**Table:**

	*x*	*y*	*z*	*U*_iso_*/*U*_eq_	
C1	0.5240 (3)	0.5539 (6)	0.6801 (5)	0.0531 (12)	
H1	0.5027	0.4792	0.7312	0.064*	
C2	0.4619 (4)	0.6615 (7)	0.6053 (6)	0.0591 (14)	
H2	0.3973	0.6590	0.6052	0.071*	
C3	0.4949 (4)	0.7726 (6)	0.5310 (5)	0.0605 (14)	
H3	0.4518	0.8430	0.4806	0.073*	
C4	0.5905 (4)	0.7819 (6)	0.5296 (5)	0.0528 (12)	
H4	0.6120	0.8597	0.4807	0.063*	
C4A	0.6539 (3)	0.6727 (5)	0.6026 (4)	0.0419 (11)	
C4B	0.7600 (3)	0.6587 (6)	0.6089 (4)	0.0431 (11)	
C5	0.7758 (3)	0.5980 (6)	0.4897 (4)	0.0465 (11)	
H5	0.7546	0.6673	0.4234	0.056*	
C6	0.8171 (3)	0.4552 (6)	0.4730 (5)	0.0522 (12)	
H6	0.8219	0.4270	0.3958	0.063*	
C7	0.8568 (3)	0.3362 (6)	0.5723 (5)	0.0509 (12)	
H7	0.8103	0.2461	0.5740	0.061*	
C8	0.8773 (3)	0.4279 (6)	0.6908 (5)	0.0494 (12)	
H8A	0.8925	0.3461	0.7554	0.059*	
H8B	0.9321	0.5013	0.6960	0.059*	
N8A	0.7945 (3)	0.5296 (5)	0.7036 (3)	0.0438 (9)	
C9A	0.6193 (3)	0.5621 (5)	0.6755 (4)	0.0445 (11)	
C10	0.8122 (4)	0.8269 (6)	0.6448 (5)	0.0585 (14)	
H10A	0.7957	0.8700	0.7158	0.088*	
H10B	0.7939	0.9074	0.5810	0.088*	
H10C	0.8798	0.8085	0.6601	0.088*	
O1	0.6992 (3)	0.2591 (4)	0.7125 (3)	0.0559 (9)	
O2	0.7235 (3)	0.4530 (5)	0.8805 (3)	0.0643 (10)	
S9	0.70986 (9)	0.43152 (14)	0.75307 (11)	0.0450 (3)	
Br	0.97557 (5)	0.23461 (9)	0.54940 (6)	0.0811 (3)	

### Atomic displacement parameters (Å^2^)

**Table:**

	*U*^11^	*U*^22^	*U*^33^	*U*^12^	*U*^13^	*U*^23^
C1	0.055 (3)	0.050 (3)	0.061 (3)	−0.002 (2)	0.027 (3)	−0.008 (2)
C2	0.048 (3)	0.053 (3)	0.080 (4)	0.004 (2)	0.023 (3)	−0.015 (3)
C3	0.062 (4)	0.049 (3)	0.069 (3)	0.017 (2)	0.010 (3)	−0.008 (3)
C4	0.065 (4)	0.040 (2)	0.055 (3)	0.006 (2)	0.017 (2)	0.002 (2)
C4A	0.054 (3)	0.034 (2)	0.041 (3)	0.000 (2)	0.017 (2)	−0.0063 (19)
C4B	0.052 (3)	0.039 (2)	0.040 (3)	−0.001 (2)	0.016 (2)	0.001 (2)
C5	0.052 (3)	0.052 (3)	0.039 (3)	0.000 (2)	0.016 (2)	0.004 (2)
C6	0.054 (3)	0.060 (3)	0.045 (3)	−0.003 (2)	0.015 (2)	−0.008 (2)
C7	0.050 (3)	0.046 (3)	0.062 (3)	0.001 (2)	0.024 (2)	−0.001 (2)
C8	0.042 (3)	0.053 (3)	0.052 (3)	0.001 (2)	0.008 (2)	0.007 (2)
N8A	0.047 (2)	0.045 (2)	0.041 (2)	−0.0008 (17)	0.0129 (18)	0.0024 (17)
C9A	0.051 (3)	0.041 (2)	0.044 (3)	0.001 (2)	0.017 (2)	−0.004 (2)
C10	0.069 (4)	0.043 (3)	0.063 (4)	−0.012 (3)	0.014 (3)	−0.004 (2)
O1	0.063 (2)	0.0424 (19)	0.066 (2)	0.0003 (15)	0.0238 (16)	0.0028 (18)
O2	0.079 (3)	0.080 (3)	0.038 (2)	0.003 (2)	0.0223 (18)	0.0037 (17)
S9	0.0546 (7)	0.0438 (6)	0.0403 (7)	0.0009 (5)	0.0187 (5)	0.0036 (5)
Br	0.0675 (5)	0.0895 (5)	0.0925 (5)	0.0267 (3)	0.0314 (3)	−0.0027 (4)

### Geometric parameters (Å, °)

**Table:**

C1—C2	1.382 (8)	C6—C7	1.487 (7)
C1—C9A	1.387 (7)	C6—H6	0.9300
C1—H1	0.9300	C7—C8	1.508 (7)
C2—C3	1.380 (8)	C7—Br	1.959 (5)
C2—H2	0.9300	C7—H7	0.9800
C3—C4	1.382 (8)	C8—N8A	1.472 (6)
C3—H3	0.9300	C8—H8A	0.9700
C4—C4A	1.390 (7)	C8—H8B	0.9700
C4—H4	0.9300	N8A—S9	1.648 (4)
C4A—C9A	1.377 (6)	C9A—S9	1.741 (5)
C4A—C4B	1.517 (6)	C10—H10A	0.9600
C4B—N8A	1.490 (6)	C10—H10B	0.9600
C4B—C5	1.513 (7)	C10—H10C	0.9600
C4B—C10	1.535 (7)	O1—S9	1.435 (3)
C5—C6	1.308 (7)	O2—S9	1.440 (4)
C5—H5	0.9300		
			
C2—C1—C9A	117.1 (5)	C8—C7—Br	108.5 (3)
C2—C1—H1	121.5	C6—C7—H7	109.1
C9A—C1—H1	121.5	C8—C7—H7	109.1
C3—C2—C1	120.6 (5)	Br—C7—H7	109.1
C3—C2—H2	119.7	N8A—C8—C7	110.7 (4)
C1—C2—H2	119.7	N8A—C8—H8A	109.5
C2—C3—C4	121.5 (5)	C7—C8—H8A	109.5
C2—C3—H3	119.2	N8A—C8—H8B	109.5
C4—C3—H3	119.2	C7—C8—H8B	109.5
C3—C4—C4A	118.8 (5)	H8A—C8—H8B	108.1
C3—C4—H4	120.6	C8—N8A—C4B	116.4 (4)
C4A—C4—H4	120.6	C8—N8A—S9	117.2 (3)
C9A—C4A—C4	118.7 (5)	C4B—N8A—S9	114.9 (3)
C9A—C4A—C4B	115.0 (4)	C4A—C9A—C1	123.3 (5)
C4—C4A—C4B	126.3 (4)	C4A—C9A—S9	110.7 (4)
N8A—C4B—C5	110.4 (4)	C1—C9A—S9	125.9 (4)
N8A—C4B—C4A	104.5 (4)	C4B—C10—H10A	109.5
C5—C4B—C4A	109.6 (4)	C4B—C10—H10B	109.5
N8A—C4B—C10	109.4 (4)	H10A—C10—H10B	109.5
C5—C4B—C10	110.6 (4)	C4B—C10—H10C	109.5
C4A—C4B—C10	112.1 (4)	H10A—C10—H10C	109.5
C6—C5—C4B	124.8 (5)	H10B—C10—H10C	109.5
C6—C5—H5	117.6	O1—S9—O2	114.9 (2)
C4B—C5—H5	117.6	O1—S9—N8A	111.5 (2)
C5—C6—C7	122.7 (5)	O2—S9—N8A	110.6 (2)
C5—C6—H6	118.6	O1—S9—C9A	112.5 (2)
C7—C6—H6	118.6	O2—S9—C9A	111.4 (2)
C6—C7—C8	110.5 (4)	N8A—S9—C9A	94.2 (2)
C6—C7—Br	110.6 (3)		
			
C9A—C1—C2—C3	−0.7 (8)	C10—C4B—N8A—C8	−89.4 (5)
C1—C2—C3—C4	−0.8 (8)	C5—C4B—N8A—S9	−109.8 (4)
C2—C3—C4—C4A	1.9 (7)	C4A—C4B—N8A—S9	7.9 (4)
C3—C4—C4A—C9A	−1.4 (7)	C10—C4B—N8A—S9	128.2 (4)
C3—C4—C4A—C4B	177.0 (4)	C4—C4A—C9A—C1	−0.1 (7)
C9A—C4A—C4B—N8A	−3.8 (5)	C4B—C4A—C9A—C1	−178.7 (4)
C4—C4A—C4B—N8A	177.8 (4)	C4—C4A—C9A—S9	177.2 (3)
C9A—C4A—C4B—C5	114.5 (4)	C4B—C4A—C9A—S9	−1.4 (5)
C4—C4A—C4B—C5	−63.9 (6)	C2—C1—C9A—C4A	1.2 (7)
C9A—C4A—C4B—C10	−122.3 (5)	C2—C1—C9A—S9	−175.7 (4)
C4—C4A—C4B—C10	59.3 (6)	C8—N8A—S9—O1	−33.8 (4)
N8A—C4B—C5—C6	−2.3 (7)	C4B—N8A—S9—O1	108.2 (3)
C4A—C4B—C5—C6	−117.0 (5)	C8—N8A—S9—O2	95.3 (4)
C10—C4B—C5—C6	118.9 (5)	C4B—N8A—S9—O2	−122.7 (3)
C4B—C5—C6—C7	−2.0 (8)	C8—N8A—S9—C9A	−150.0 (3)
C5—C6—C7—C8	−21.9 (7)	C4B—N8A—S9—C9A	−8.0 (3)
C5—C6—C7—Br	−142.1 (4)	C4A—C9A—S9—O1	−110.0 (3)
C6—C7—C8—N8A	49.3 (5)	C1—C9A—S9—O1	67.2 (5)
Br—C7—C8—N8A	170.7 (3)	C4A—C9A—S9—O2	119.3 (4)
C7—C8—N8A—C4B	−57.5 (5)	C1—C9A—S9—O2	−63.5 (5)
C7—C8—N8A—S9	84.0 (4)	C4A—C9A—S9—N8A	5.3 (4)
C5—C4B—N8A—C8	32.5 (5)	C1—C9A—S9—N8A	−177.5 (4)
C4A—C4B—N8A—C8	150.3 (4)		
